# Innovations in Positron Emission Tomography and State of the Art in the Evaluation of Breast Cancer Treatment Response

**DOI:** 10.3390/jcm13010154

**Published:** 2023-12-27

**Authors:** Luigi Castorina, Alessio Danilo Comis, Angela Prestifilippo, Natale Quartuccio, Stefano Panareo, Luca Filippi, Serena Castorina, Dario Giuffrida

**Affiliations:** 1 Nuclear Medicine Outpatient Unit, REM Radiotherapy Srl, Via Penninanzzo 11, 95029 Viagrande, Italy; alessiodanilo.comis@gmail.com; 2 Department of Oncology, IOM Mediterranean Oncology Institute, Via Penninanzzo 7, 95029 Viagrande, Italy; angela.prestifilippo@grupposamed.com (A.P.); dario.giuffrida@grupposamed.com (D.G.); 3 Nuclear Medicine Unit, Ospedali Riuniti Villa Sofia-Cervello, 90146 Palermo, Italy; n.quartuccio@villasofia.it; 4 Nuclear Medicine Unit, Oncology and Haematology Department, University Hospital of Modena, 41124 Modena, Italy; panareo.stefano@aou.mo.it; 5 Nuclear Medicine Unit, Department of Oncohaematology, Fondazione PTV Policlinico Tor Vergata University Hospital, Viale Oxford 81, 00133 Rome, Italy; luca.filippi@ptvonline.it; 6 Nuclear Medicine Unit, Azienda Ospedaliero Universitaria Policlinico “G. Rodolico-San Marco”, 95123 Catania, Italy

**Keywords:** PET/CT, PET/MR, FDG, breast cancer, neoadjuvant chemotherapy, response to therapy

## Abstract

The advent of hybrid Positron Emission Tomography/Computed Tomography (PET/CT) and PET/Magnetic Resonance Imaging (MRI) scanners resulted in an increased clinical relevance of nuclear medicine in oncology. The use of [18F]-Fluorodeoxyglucose ([18F]FDG) has also made it possible to study tumors (including breast cancer) from not only a dimensional perspective but also from a metabolic point of view. In particular, the use of [18F]FDG PET allowed early confirmation of the efficacy or failure of therapy. The purpose of this review was to assess the literature concerning the response to various therapies for different subtypes of breast cancer through PET. We start by summarizing studies that investigate the validation of PET/CT for the assessment of the response to therapy in breast cancer; then, we present studies that compare PET imaging (including PET devices dedicated to the breast) with CT and MRI, focusing on the identification of the most useful parameters obtainable from PET/CT. We also focus on novel non-FDG radiotracers, as they allow for the acquisition of information on specific aspects of the new therapies.

## 1. Introduction

The technological evolution of Positron Emission Tomography (PET) has led to the creation of hybrid PET/Computed Tomography (CT) and PET/Magnetic Resonance Imaging (MRI) scanners and highly sensitive PET tomographs with three or up to five rings. PET imaging has quickly established itself as a fundamental tool in the management of patients suffering from oncological pathologies.

Due to its high diagnostic sensitivity (picomolar, more than 100 times smaller than standard molecular imaging), PET/CT is a powerful imaging tool for quantifying distinct biological processes [1].

Particularly, [^18^F]Fluorodeoxyglucose ([^18^F]FDG) has proven to be the most effective radiopharmaceutical in the metabolic evaluation and molecular imaging of most cancers.

Breast cancer (BC) became the most common type of cancer in the world among women in 2020, surpassing lung cancer, with an estimated 2.3 million new cases (representing 11.7% of all cancer cases). It also stood as the fifth leading cause of cancer mortality worldwide, accounting for 685,000 deaths that year [2,3].

The incidence rate of BC is rapidly increasing due to the increased precision of the diagnostic techniques, the population undergoing screening, the elderly population, and risk factors. Early diagnosis and advancements in personalized treatments have contributed to a reduction in mortality in developed countries in the last few decades [4,5]; nonetheless, BC-related deaths are expected to increase in the future [6], with about 30% of patients with loco-regional disease developing metastases, making the disease incurable despite improvements in treatments [7].

The disease is characterized by remarkable heterogeneity, which is expressed in distinct biological variables that influence the response to therapy and, ultimately, the prognosis [8].

The main prognostic factors for BC are tumor size, axillary nodal status, tumor pathology, tumor grade, peritumoral lymphatic vessel and vascular invasion, hormonal receptor status, proliferation markers, age, and ethnicity, among others [9,10].

BC molecular subtypes are defined based on the state of the estrogen receptors (ERs), progesterone receptors (PRs), and human epidermal growth factor 2 receptors (HER2s). Based on this different receptor expression, four different molecular subtypes are classified: luminal A (ER-positive, PR-positive over 20%, HER2-negative, proliferation index Ki 67 less than 20%); luminal B (hormone receptors positive but high proliferative index); HER2-positive (hormone receptors negative, overexpression of HER2s); and triple negative (TN, no expression of hormone receptors or HER2s).

This classification underlies the prognosis and management of the disease [11,12].

In patients with locally advanced BC (stage II/III), neoadjuvant chemotherapy (NAC) is well codified in international guidelines.

Overall, NAC compared with mastectomy followed by adjuvant chemotherapy does not change patient outcome, but pathologic complete response (pCR) is a powerful marker for a better outcome. However, only 29.7% of patients undergoing NAC achieve a pCR [13]. When compared to women without a pCR, patients who had obtained a pCR had an improved 5-year disease-free survival rate of 87% and a 5-year overall survival rate of 89% [14,15,16].

Thus, early control of the effectiveness of NAC allows for the maintenance or refinement of the chemotherapy regimen or the reduction in the toxicities of ineffective chemotherapy [17].

Therefore, it becomes necessary to have a tool as precise as possible for evaluating the response to therapy. [^18^F]FDG PET/CT is useful in the initial staging, restaging, evaluation of the treatment response, and prediction of the prognosis of BC [18]. [^18^F]FDG PET/CT has higher sensitivity than conventional imaging for the diagnosis of distant metastases [19,20].

Two meta-analyses that used histopathology as the standard of care evaluated the clinical validity of serial [^18^F]FDG PET/CT scans to track therapeutic responses to NAC. The results showed a pooled sensitivity of 82–86% and a specificity of 72–79% [21,22,23,24,25].

The aim of our review is to propose the results of the most recent studies (published especially in the period 2018–2021) on treatment response evaluation in BC patients who received any form of therapy.

## 2. Materials and Methods

Due to the narrative nature of this review, the literature search and selection of articles were not performed by means of a systematic protocol. The PubMed/Medline database was used for the literature search. PubMed was favored over clinical trial databases due to its wide range of biomedical literature beyond the constraints of clinical trials. Indeed, clinical trial databases (such as ClinicalTrials.gov) concentrate on ongoing and finished clinical trials, whereas PubMed encompasses a broad spectrum of biomedical content, spanning research articles, reviews, case studies, and beyond. When aiming for a comprehensive understanding of a topic, PubMed’s broader range of content may be better suited for a review.

The PubMed/Medline database was searched by two authors using combinations of the following terms and their derivatives: “PET”, “breast cancer”, and “response to therapy”.

We selected 330 studies in English up to 26 November 2023 (the day of the literature search). Twenty-nine of these were excluded as preclinical studies and twenty-five as inherent to radiomics. In addition, we reviewed further reviews and meta-analyses to retrieve additional articles. Accordingly, a total of 876 studies were reviewed, and 127 bibliographic entries were finally included for the discussion in the present review.

The chronological distribution of the studies is presented in Table 1.

## 3. Results

For ease of presentation, the selected studies will be presented grouped by macro-topics.

### 3.1. [^18^F]FDG PET/CT in Assessing BC’s Response to Therapy

The European Society for Medical Oncology (ESMO) recommends imaging of the chest, abdomen, and bone for staging metastatic BC (MBC) and, afterwards, imaging of target lesions every 2–4 months while receiving treatment to evaluate response to treatment. However, ESMO does not specify the appropriate modality or response criteria [26].

In 2018, two Japanese studies supported the complementarity of the “Response Evaluation Criteria in Solid Tumors” (RECIST) and the “PET Response Criteria in Solid Tumors” (PERCIST) in the assessment of response to therapy in BC, highlighting a high specificity and negative predictive value (NPV) for CT and a high sensitivity and positive predictive value (PPV) for PET/CT [27,28]. Furthermore, PERCIST’s accuracy demonstrated good accuracy in predicting post-NAC pCR in the HER2-positive and triple-negative (TN) phenotypes, but poor accuracy in luminal A and B.

In a systematic review, Helland et al. support the supremacy of PERCIST based on [^18^F]FDG PET/CT over contrast-enhanced CT (CE-CT) due to greater repeatability, less inter-observer variability, and the ability to detect bone lesions and distinguish between tumor activity and response to therapy. Nevertheless, major guidelines do not recommend [^18^F]FDG PET/CT-based PERCIST in evaluating treatment response in BC. The authors further suggest that, even in the absence of cost-effectiveness analyses, [^18^F]FDG PET/CT’s earlier and more accurate assessment over CE-CT in identifying non-response to therapy makes PET/CT cheaper than CE-CT, avoiding the costs of ineffective therapies [29].

The early predictive usefulness of the European Organization for Research and Treatment of Cancer (EORTC) and the PERCIST criteria for the pCR and prognosis of BC patients undergoing NAC have also been compared. The major findings in a study were that metabolic changes can accurately predict long-term outcomes (accuracy 74–75%) and that PERCIST may be more useful than EORTC in detecting progression. Furthermore, PET parameters such as the Δ% of the peak of the standardized uptake value normalized for lean body mass (Δ%SULpeak), the Δ%SUVmax, the Δ% of the metabolic tumor volume (Δ%MTV), and the Δ% of the total lesion glycolysis (Δ%TLG) could predict NAC response but did not correlate with prognosis. The results for sensitivity, specificity, PPV, NPV, and accuracy to predict pCR were 69.7%, 76.3%, 62.2%, 81.8%, and 73.9% for EORTC, and 69.7%, 77.9%, 63.9%, 82.1%, and 75.0% for PERCIST [30]. Table 2 summarizes the main features of the EORTC and PERCIST criteria.

The differences in PERCIST readings between prone and supine scan orientations in [^18^F]FDG PET/CT of the breast were examined in an original study. There were no statistically significant differences in PERCIST measurements for predicting pCR for any of the investigated PET parameters (SUVpeak, SUVmax, SULpeak, and SULmax) [31].

In another study, Hulikal et al. demonstrated that PET/CT was more accurate (87%) than clinical assessment (39%) and CT (56%) for response evaluation [32].

A meta-analysis by Han and Choi [33] reported that overall survival (OS) and disease recurrence can be predicted by the PET metabolic response during and after NAC. In particular, a %ΔSUV of 74% in breast tumors was a predictor of disease-free survival (DFS) [34]. Furthermore, according to the meta-analysis, there were no differences in the prognostic values of interim and post-treatment PET scans. The timing of the interim assessment by means of PET is typically carried out after two cycles of therapy (followed by PET after one cycle) and produces significant prognostic values. Notably, the cutoff values for each PET parameter varied substantially between studies in more than half of the included trials because they were data-dependent.

The usefulness of [^18^F]FDG PET/CT in assessing the response to treatment has been observed in many subsequent investigations [35,36,37,38]. Kwon et al. assessed the correlations between the histochemical profiles and the metabolic information of [^18^F]FDG PET to better characterize BC subtypes [39]. They discovered that molecular alteration profiles determined by immunohistochemistry data substantially correlated with the metabolic activity of BC as evaluated by [^18^F]FDG PET/CT. Tumor SUV was substantially correlated with tumor size; expression levels of p53, Ki-67, and EGFR; and nuclear grade (*p* < 0.001). Contrarily, the expression of estrogen receptors (*p* < 0.001) and progesterone receptors (*p* < 0.001) was inversely correlated with tumor SUV.

### 3.2. PET/CT Parameters

The PERCIST criteria, used in the research studies reported above, are based on SUVSUL. More recently, research has investigated volume-based metabolic variables obtained with [^18^F]FDG PET/CT, which produced more accurate indicators of both NAC response and prognosis [40,41,42,43].

In BCs treated with chemotherapy, the baseline MTV and ΔSUVmean can accurately predict pCR. In BCs treated with preoperative chemotherapy, the predictive value of this combination is independent and stronger than other clinical variables such as tumor size, tumor grade, Ki67 levels, and BC subtypes [44].

Humbert et al. studied the blood flow (BF) and metabolism of breast cancers using a single [^18^F]FDG PET combining a dynamic, short first-pass PET acquisition with a standard delayed PET acquisition, observing that these two functional pathways offer various and complementary biological information. BC subtypes responded to NAC in a wide variety of ways. Namely, a marked decline in BF in HER2-positive tumors after the first cycle of trastuzumab (first reported in the literature) was shown to be an important finding because trastuzumab has a strong antiangiogenic effect. The average BF decrease varies greatly depending on the tumor and is modest in TN BC and luminal/HER2-negative BC, indicating biological heterogeneity. BC subtypes responded to NAC in a wide variety of ways [45].

### 3.3. Dedicated PET Scanner

There are two types of scanners available for dedicated breast PET (dbPET) imaging, a recently established technique for identifying breast disease: opposite and ring. After NAC, dbPET has demonstrated utility in predicting the presence of residual invasive and non-invasive breast tumors. In the latter, due to the relatively low SUVmax associated with decreased tumor cell density and metabolic activity after NAC, the tumor-to-normal tissue ratio (TNR) can be a better indicator of pathological response than SUVmax. The sensitivity, specificity, and accuracy of dbPET-TNR were 60%, 100%, and 70.2%, respectively, when the cutoff was 1.6 [46,47].

dbPET has high photon sensitivity and may increase its spatial resolution by placing the detector near the breast, minimizing respiratory movements, and employing smaller detection units by applying reconstruction techniques that are different from those used for whole-body (WB) PET. dbPET was found to be the best predictor of pCR after NAC in a 2021 study with breast cancer patients treated with NAC undergoing WBPET, dbPET, and MRI. The sensitivity and specificity of dbPET were 85.7% and 72.7%; those of WB PET were 71.4% and 77.3%, respectively; and those of MRI were 100% and 50%, respectively [48].

Furthermore, high-resolution positron emission mammography (PEM) systems produce 3D breast images with an in-plane spatial resolution of 2 mm and have emerged as breast-specific PET devices, being more sensitive than conventional PET scanners. Kalinyak et al. [49], in a study of 109 primary invasive breast tumors with a mean tumor size of 1.6 ± 0.8 cm, found that the detection rates obtained with PEM and conventional PET/CT were 95 and 87%, respectively (*p* < 0.029). Furthermore, Noritake et al. [50] showed that [^18^F]FDG PEM outperformed conventional WB [^18^F]FDG PET/CT in assessing NAC response, identifying a significant difference in PEM SUVmax between patients with pCR and those without pCR.

### 3.4. PET vs. MRI

In a 2017 meta-analysis [51], the diagnostic performances of MRI and PET/CT for the assessment of BC response to NAC were comparable with high sensitivities (0.79 vs. 0.87) and specificities (0.82 vs. 0.85). Nevertheless, if we exclude CE-MRI, PET/CT is more sensitive than conventional MR imaging, with a sensitivity, respectively, of 0.88 vs. 0.74 and a specificity of 0.82 for both imaging methods. PET/CT has been shown to be comparable to functional MRI with a lower pooled sensitivity (0.78 vs. 0.88), better pooled specificity (0.92 vs. 0.82), and similar AUC (0.93 vs. 0.89). Moreover, when evaluating the responses between one and three cycles of NAC, PET/CT is preferable to MRI because it has a significantly greater pooled specificity (0.94 vs. 0.83) and a comparable pooled sensitivity (0.71 vs. 0.73). Conversely, after three cycles of NAC, the combined sensitivity, specificity, and AUC of PET/CT and MRI were very similar.

The sensitivity of MRI was higher than that of PET/CT, according to the pooled data of the 13 studies that made up a 2018 meta-analysis [52], but the specificity was lower (0.69 vs. 0.78). Conversely, Liu et al., in a systematic review and meta-analysis published in 2019 [53], showed that [^18^F]FDG PET/CT has a greater sensitivity and MRI has a higher specificity in assessing pCR in breast cancer patients. As a result, the combined use of these two imaging modalities may greatly enhance diagnostic performance when evaluating pCR after NAC.

### 3.5. PET/CT and PET/MRI

Dedicated breast exams as well as WB exams may benefit from the use of the innovative hybrid imaging technology known as PET/MRI. When used with multiple MRI parameters, breast PET/MRI has shown potential in minimizing unnecessary biopsies that would be advised based on their current standard dynamic contrast-enhanced (DCE)-MRI. On the one hand, it is improbable that PET/MRI breast imaging will become a common practice in clinical settings due to the imaging processing and reading times involved. On the other hand, breast PET/MRI may be more crucial before and during neoadjuvant therapy, where multiple layers of imaging parameters may be converted into radiomic data that may increase the precision of breast cancer treatments. It may also be more crucial during local staging, where the enhanced assessment of the axilla, potentially provided by PET/MRI, may eventually eliminate the need for axillary lymph node tissue sampling. For BC patients who require staging or post-treatment follow-up, PET/MRI performs better than PET/CT while using a significantly lower radiation dosage [54].

Cho et al. also argued that patients with BC can use [^18^F]PET/MRI to predict non-pCR after the first cycle of NAC. The addition of MRI parameters to the PET parameters has the potential to increase sensitivity. In their study, PET parameters (including SUVmax, MTV, and TLG) and MRI parameters (including washout proportion and signal enhancement ratio (SER)) were examined. TLG30%° had a sensitivity of 63.2% (12/19) in predicting non-pCR, and SER had a sensitivity of 84.2% (16/19). Specificity was 71.4% (5/7) and sensitivity was 100% (19/19) when the combined TLG30% and SER criteria were used [55].

### 3.6. Subtypes of Breast Cancer

#### 3.6.1. Luminal Subtype

Different biological entities make up BC. More than half of breast cancers in women have the luminal/HER2-negative subtype, which is characterized by the expression of hormone receptors (HRs) but not by an overexpression of human HER2. Despite having less chemosensitivity than other subtypes, it has a more favorable outcome. pCR is rarely attained in this subtype.

In luminal A-like BC, [^18^F]FDG uptake may be affected by physiological hormonal changes during the menstrual cycle: in premenopausal individuals with luminal A-like tumors, SUVmax levels during the periovulatory–luteal phase were considerably greater than during the follicular phase [56].

In a study of patients with localized luminal/HER2-negative breast cancer for whom breast-conserving surgery was initially not feasible and NAC was prescribed to reduce tumor, Grapin et al. found that breast size (women with a total breast volume higher than 496 mL, quantified precisely on the coregistration CT of the PET system) and tumor chemosensitivity (positive metabolic response after the first cycle of treatment, defined by ΔMTV-17%) were the two main predictive imaging parameters of breast-conserving surgery. These two factors were combined to create three patient groups with varied chances of getting breast-conserving surgery. According to their findings, the likelihood of undergoing breast-conserving surgery steadily improved from 29% in the group with the worst prognosis (low total breast volume and poor tumor shrinking) to 82% in the group with the best prognosis (high total breast volume and significant tumor metabolic shrinkage) [57].

For post-menopausal women with ER+/HER2− BC, neoadjuvant endocrine therapy (NET) is a well-known therapeutic option. Early metabolic responses can be more informative than morphological responses. According to research by Boughdad et al., [^18^F]FDG PET/CT may become a simple and affordable “surrogate marker” to track tumor response, particularly in post-menopausal women with ER-rich/HER2− BC who may benefit from NET therapy [58].

According to the findings of a different study, PET imaging might be a good method for choosing the initial therapy strategy and maybe maximizing the possibility of breast preservation in patients with HR-positive, HER2-negative (particularly luminal B-like type) BC [59,60].

#### 3.6.2. HER2-Positive

HER2 overexpression is observed in 15% to 25% of all breast cancers [61].

HER2(+) BC has been recognized as a more aggressive early-stage BC than other subtypes. HER2 overexpression is associated with early tumor cell dissemination to secondary organs, causing an increased likelihood of metastatic disease, which is responsible for the majority of cancer morbidity and mortality [62].

Re-evaluating HER2 expression in metastatic tumors is important since there is a wide range of HER2 expression between distant metastatic lesions and original tumors (4.9–17.7%). Furthermore, HER2 expression may change over time after cancer develops, necessitating ongoing HER2 testing. However, repeated biopsies are uncomfortable for the patient. This restriction can be bypassed by non-invasive HER2 expression analysis using specific radioisotopes [63].

Patients with HER2-positive, early-stage breast cancer now have a much better prognosis thanks to the development of HER2-targeted agents. With no chemotherapy, neoadjuvant dual HER2 blockade with trastuzumab and pertuzumab has resulted in remarkable pathological complete response rates (20.5 to 36.3%). Numerous studies conducted since 2012 have evaluated de-escalation strategies for patients with HER2-positive, early-stage breast cancer [64,65,66,67,68,69,70,71,72,73].

The effectiveness (in terms of pCR and 3-year DFS) of dual HER2 blockade with trastuzumab and pertuzumab with or without endocrine therapy in patients with HER2-positive, early-stage breast cancer who were [^18^F]FDG PET responders was examined in a multicenter, randomized, open-label, non-comparative phase-2 trial involving 45 hospitals in Spain, France, Belgium, Germany, the UK, Italy, and Portugal (PHERGain). A total of 37.9% of the patients who were [^18^F]FDG PET responders achieved a pCR in the breast and axilla (a proportion greater than the historical rate) and, therefore, may be able to decline chemotherapy as part of their treatment [74].

The predictive efficacy of early PET evaluation in patients with early-stage HER2-positive breast cancer was examined in the open-label, randomized, phase II AVATAXHER trial. The authors noted that early PET allowed the identification of HER2-positive BC patients who responded poorly to neoadjuvant treatment. Additionally, they noticed that the pCR rate was increased by the addition of bevacizumab to the neoadjuvant scheme (docetaxel plus trastuzumab). However, this increase in the pCR rate did not result in a long-term increase in DFS [75].

#### 3.6.3. Triple-Negative BC

TN breast cancer has no expression of ER, PR, or HER-2.

TNBC makes up between 15 and 20% of all invasive breast cancers and is distinguished from other subtypes by its ductal histology, high mitotic rates, and early lymph node involvement. TNBC patients face a dismal prognosis because of their aggressive nature and resistance to hormone and targeted therapy.

The median OS for metastatic TNBC is only 1–1.5 years [76].

The typical pharmacological strategy for treating TNBC consists in using NAC in combination with anthracyclines and the mitotic inhibitor taxanes.

The “triple-negative paradox” is a phenomenon where TNBC is particularly sensitive to NAC despite its inherent aggressiveness. Unfortunately, patients who do not reach pCR have a high recurrence rate. For this reason, it would be important to identify a biomarker able to predict the response to NAC, thus avoiding the use of ineffective drugs and optimizing therapy management. Therefore, it has been suggested that a decrease in [^18^F]FDG uptake following two cycles of neoadjuvant chemotherapy is a potent indicator of patients’ outcomes [77].

Early change in tumor [^18^F]FDG uptake during NAC (ΔSUVmax) was highly predictive of pCR in TNBC patients. Conversely, baseline SUVmax alone was not predictive of pCR [78].

A factor that has been found to be very important in the response to treatment is tumor heterogeneity, which is greater in TNBCs than in other BCs. A study by Gong et al. proposed the heterogeneity index (HI), evaluated by the maximum [18F]FDG uptake divided by the minimum [^18^F]FDG uptake across the metastatic lesions, as a new parameter to assess the value of heterogeneity among metastatic lesions on pretreatment PET/CT. The median progression-free survival (PFS) for patients with high HI (>1.9) was 7.8 months, which was substantially less than the median PFS for individuals with low HI (<1.9), which was 10.9 months (*p* = 0.049). Patients with high HI-T also had a shorter median OS, although this difference was not statistically significant (19.9 vs. 25.6 months, *p* = 0.597) [79].

The poly (adenosine diphosphate-ribose) polymerase (PARP) inhibitor olaparib, a novel drug class that has shown promise in treating tumors containing germline BRCA1/2 mutations, was the focus of the Italian study OLTRE, which looked primarily at the biological, immunologic, and genetic changes in gBRCA-wild-type TNBCs. Olaparib may be useful in treating both gBRCA-mutant tumors and gBRCA-wild-type TNBCs, according to the considerable decrease in tumor clinical dimension and SUVmax that was seen in the study [80].

#### 3.6.4. Response to Therapy in Invasive Lobular Breast Cancer

All existing imaging modalities, including [^18^F]FDG PET, have difficulty in visualizing invasive lobular breast cancer (ILC), a subtype of breast cancer that makes up 10%–15% of primary breast malignancies. A correlation between the changes in the corrected SUVmax and the ILC tumor response was demonstrated in a study using ^18^F-Fluciclovine, a radiotracer used to image amino acid transport in tumors. Compared to invasive ductal carcinoma (IDC), ILC had a lower rate of pCR after NAC [81].

ILC is harder to find with imaging techniques, including mammography, ultrasound, MRI, and [^18^F]FDG PET/CT, because of the decreased cellular density per unit volume. ILC (both primary and metastatic forms) has lower SUVs on [^18^F]FDG PET than IDC tumors. ILC patients benefit more from ER-targeting PET tracers since they are almost always (95%) estrogen receptor (ER)-positive [82].

### 3.7. Response to Therapy in Metastatic Breast Cancer

#### 3.7.1. Assessment of Response to Therapy in BCs with Axillary Metastases

A full axillary (Ax) response to NAC occurs in around 40% of BC patients with axillary lymph node metastases. In certain studies, individuals with HER2 positivity who received trastuzumab had an Ax-pCR rate of more than 80%. In the past, patients with axillary lymph node metastases were routinely treated after NAC with axillary lymph node dissection (ALND). However, recent studies have reported that the morbidity of axillary dissection can be avoided by employing sentinel lymph node biopsy (SLNB) for patients showing a full axillary response following NAC. Therefore, the optimal surgical care of axillary lymph nodes can be chosen with the help of a detailed evaluation of the post-NAC axillary response.

According to Koolen et al., a relative decrease in SUVmax was significantly higher in patients with Ax-pCR than in those without it, and a relative decrease in SUVmax (more than 60%) on [^18^F]FDG PET/CT after two cycles of NAC could better predict Ax-pCR [83].

In patients with node-positive breast cancer after NAC, the use of subtype-guided [^18^F]FDG PET/CT could predict nodal response and personalize axillary surgery [84].

Samiei et al. conducted a systematic review and meta-analysis to assess the diagnostic performance of axillary ultrasonography, breast MRI, and whole-body [^18^F]FDG PET-CT after neoadjuvant systemic therapy (NST) for axillary response in clinically node-positive breast cancer patients. They showed that axillary ultrasound, breast MRI, and whole-body PET-CT can correctly identify residual axillary lymph node disease following NST only in 77%, 78%, and 78% of the node-positive patients, respectively. These numbers are significantly lower for axillary pCR determination (50%, 58%, and 49%, respectively, for axillary ultrasound, breast MRI, and whole-body PET/CT). Hence, they concluded that imaging cannot contribute to or replace axillary procedures following NST in clinically node-positive breast cancer [85].

On the other hand, Kim et al. discovered that for restaging, MR imaging, and PET/CT, the NPV for excluding advanced ALN metastases was high. Therefore, in patients with invasive ductal carcinoma undergoing NAC, breast MRI and PET/CT during restaging can serve as instruments to guide axillary surgery for a less invasive approach [86].

Turan et al. compared the post-NAC results of US, MRI, and [^18^F]FDG PET/CT in the evaluation of the persistence or otherwise of the disease in axillary lymph nodes diagnosed as metastatic at the time of diagnosis. The obtained data on sensitivity, specificity, PPV, and NPV were, respectively, 59%, 82%, 82%, and 60% for US (the highest); 36%, 77%, 73%, and 42% for MRI; and 47%, 76%, 73%, and 52% for [^18^F]FDG PET/CT. When US and PET were used together, the specificity and PPV were 100%, indicating that ALND is the preferred surgical option if US and PET together confirm the presence of metastatic axillary lymph nodes following NAC. Otherwise, with a complete axillary pathological response, the most correct surgical choice is SLNB [87].

The axillary response to NST is subtype-dependent, and patients with HER2-positive and TN BCs are more likely to achieve an axillary pathologic full response than patients with estrogen receptor-positive breast cancer [88].

It is to be considered that, in comparison to patients with residual axillary disease, patients with axillary pCR had better OS (85% vs. 55%) and DFS (83% vs. 58%) rates [89].

For patients with ER-positive/HER2-negative breast cancer, there was not a significant distinction in PET parameters between those with axillary residual disease and those with axillary pathologic full response. In the combined HER2-positive/TN subgroup, SUVmax was significantly lower in patients without residual axillary disease and in patients with clinically node-positive disease. Therefore, in HER2-positive and TN BC patients, SUVmax and SUVmean could predict axillary response [90].

#### 3.7.2. Assessment of Response to Therapy in BC with Bone Metastases

At least 70% of patients with metastatic breast cancer experience bone metastases, which are seriously detrimental to their health. Prospective evidence suggests that [^18^F]FDG PET/CT is better than a CT scan for the investigation of malignancy in patients with suspicion of bone metastases [91,92].

[^18^F]FDG, due to its higher uptake, is a more sensitive radiotracer than [^18^F]NaF in detecting lytic bone metastases, but bone scanning and [^18^F]NaF PET are more effective in detecting osteoblastic metastases [93].

In a meta-analysis, it was found that [^18^F]FDG PET/CT had the highest sensitivity compared to CE-CT (89.7% vs. 72.9%) in depicting bone metastases [94].

Koizumi et al. proposed a novel biomarker: a summation of the TLG of active bone lesions above a cutoff level. By choosing 4.0 as the SUV cutoff value, they obtained high specificity (91%) and sensitivity (97%). Whole skeletal TLG was compared with PERCIST or EORTC only for bone, and an extremely good agreement was found [95].

An interesting study by Azad et al. compared SUVmax with first-order statistical features from [^18^F]FDG PET/CT before and after endocrine treatment for BC bone metastases. They showed that the determined first-order statistical features from [^18^F]NaF and [^18^F]FDG PET—related to volume and heterogeneity, including volume-based parameters (MTV) and heterogeneity parameters (entropy, uniformity, skewness, and kurtosis)—may be more valuable than SUVmax in predicting treatment response and survival. Moreover, some heterogeneity parameters with [^18^F]NaF may be able to distinguish between an increase in SUVmax caused by a flare phenomenon and one caused by disease progression. No first-order parameters using [^18^F]FDG PET/CT were more accurate than SUVmax, despite the fact that total lesion metabolism and skewness were related to OS and PFS, respectively [96].

In another previous study, Azad et al. demonstrated that the measurement of [^18^F]NaF flux (Ki) in breast cancer bone metastases using static [^18^F]NaF PET/CT with venous blood sample counts is more effective than semiquantitative SUV measures in distinguishing progressive disease (PD) from non-PD. The determination of Ki, nonetheless, requires the acquisition, for each patient, of two venous samples at 55 and 85 min after the administration of [18F]fluoride and the measurement of a blood share in a gamma counter [97].

### 3.8. Assessment of Responses to New Therapies

In the attractive challenge of personalized medicine, [^18^F]FDG PET/CT is also increasingly becoming a helpful imaging tool for evaluating the response of BC to new targeted therapies and helping clinicians identify patients for whom therapy should be continued or intensified and those for whom it should be stopped or modified.

The effectiveness of [^18^F]FDG PET/CT was investigated in a study focusing on the response to Palbociclib, a novel targeted medication, in a group of metastatic ER-positive and HER2-negative BC patients. The better ability to demonstrate the response to new therapies by metabolic imaging compared to morphological imaging depends on the fact that, unlike typical chemotherapies, which exhibit a cytotoxic impact by means of tumor cell death, targeted therapies such as Palbociclib have a cytostatic effect (they stop tumor development). Therefore, a better imaging technique for evaluating the efficacy of targeted therapies must take tumor cell viability into account instead of only measuring changes in tumor diameters [98].

Everolimus, a rapamycin derivative, binds to mTORC1 and inhibits m-TOR. Patients with locally advanced and metastatic breast cancer who use everolimus and an endocrine agent have an increase in PFS. Moreover, the early identification of individuals who will not benefit from this is crucial since this association is characterized by a high risk of adverse events and a related high cost. Two early (3 months after therapy) ∆SUV% thresholds that can be used to identify HR+ HER2− mBC patients who would benefit or survive over the long term while receiving everolimus–exemestane treatment were found in a recent study. In particular, patients with a decrease in ∆SUV% of less than 28.8% had a significantly better 10-month PFS of 63.2% vs. 16.7% (*p* = 0.005), whereas patients with a ∆SUV% of more than 53.8% had a better 36-month OS of 82.5% vs. 45.9% (*p* = 0.048) [99].

The PEARL study (PET imaging as a biomarker of Everolimus Added value in hormone-Refractory post-menopausaL women) showed that the absence of [^18^F]FDG PET/CT metabolic response and persistent ctDNA detection after only 14 days of treatment with the EXE-EVE combination was related to a lower likelihood of benefit for patients with ER+ HER2−, non-steroidal aromatase inhibitor-resistant metastatic breast cancer [100].

### 3.9. Assessment of Response to Therapy and New Radiotracers

#### 3.9.1. Receptor Tracers

Currently, despite the well-known molecular heterogeneity of breast cancer and the discovery of significant mutations that distinguish various subtypes, the hormone receptors ER, PR, and HER2 are the only clinically relevant biomarkers and proven therapeutic targets. The receptor status can change as the disease progresses or in response to therapy; however, several invasive biopsies are not always feasible and, occasionally, may not accurately reflect the disease. Hence, new nuclear imaging agents that enable the specific identification of receptor expression may help to enhance patient care. Molecular imaging with receptor tracers can reveal information about the entire body and identify any changes in receptor expression as a disease progresses, guiding clinicians to choose the most effective treatments for each patient and assess early responses in order to reduce wasteful therapies [101].

16α-[^18^F]-fluoroestradiol PET/CT ([^18^F]FES-PET/CT) is a clear example of a non-invasive imaging modality that facilitates the assessment of the imaging of ER status, allowing the evaluation of treatment response [102].

In patients with metastatic invasive lobular carcinoma, a head-to-head comparison of [^18^F]FES PET/CT and [^18^F]FDG PET/CT showed that [^18^F]FES PET was superior to [^18^F]FDG in detecting metastatic disease locations, particularly osseous metastases [82].

Moreover, [^18^F]FES PET/CT has also been applied for predicting early responses to NAC [103].

He et al. [104]discovered a significant association between a longer PFS and a decrease in ΔSUVmax greater than 38%, as determined by serial imaging of [^18^F]FES PET/CT. Serial [^18^F]FES PET scans could distinguish patients with a disease that is receptive to fulvestrant as early as 28 days following therapy [104].

In a study by Boers et al. [105], [^18^F]FDG PET/CT monitored the effect of endocrinological therapy coupled with cyclin-dependent kinase (CDK) inhibitors (a family of drugs that prevents overgrowth of cancer cells) in metastatic ER + BC at baseline and after 8 weeks. High agreement between [^18^F]FES-PET and immunohistochemical ER status was found. [^18^F]FES PET heterogeneity can identify a subset of individuals who benefit from combination therapy (patients with 100% FES positivity), demonstrating that heterogeneity in BC is a biologically significant data point [105,106].

Another study with [^18^F]FES-PET/CT demonstrated that elacestrant (an investigational, non-steroidal, oral selective estrogen receptor degrader), as documented for fulvestrant, greatly lowers ER availability [107].

Another biomarker, 21-[F-18]-fluoro-16a,17a-[(R)-(19-a-furyl methylidene) dioxy]-19-norpregn-4-ene-3,20-dione (FFNP), has been shown to have a high affinity and selectivity for PR, offering new opportunities to assess the therapeutic response to endocrine treatment or the early response to antiestrogen therapy [108].

A list of some of the main PET radiotracers used to image breast cancer is presented in Table 3.

#### 3.9.2. Indicator of Proliferation

It has been suggested that PET with thymidine analogue 3′-[^18^F]fluoro-3′-deoxythymidine ([^18^F]FLT) is a superior biomarker than [^18^F]FDG PET for measuring a patient’s therapeutic response or resistance since it allows tumor growth fractions to be quantified. In fact, the uptake of [^18^F]FLT reflects the activity of the enzyme thymidine kinase-1 (TK1). This enzyme is well known for its role in the pyrimidine salvage pathway and is an indirect indicator of cell proliferation since it is increased during the late G1/S phase of the cell cycle [77].

In a population that received an identical NAC regimen, an Italian investigation found that an early [^18^F]FLT PET scan performed after the first cycle of treatment could accurately define the pathological response in 100% of cases [109].

A prospective study that looked into the value of pretreatment and early-treatment serial FLT-PET scans in assessing OS, PFS, and pCR in the setting of NAC for breast cancer found that PFS (7.9 years vs. 3.7 years) and OS (7.5 years vs. 5.0 years) were both longer in FLT-PET responders vs. non-responders, albeit not significantly [110].

The FLT uptake pre- and post-neoadjuvant aromatase inhibitor (AI) therapy correlated well with pre- and post-therapy Ki-67 values [111].

The metabolic response on interim [^18^F]FDG PET showed, in a head-to-head comparison between [^18^F]FDG PET and [^18^F]FLT-PET, a good predictive potential for clinical response and survival outcome in patients with mBC. Additionally, interim [^18^F]FLT PET showed a worse prognostic value, which is most likely because metastatic liver and bone lesions had poorer [^18^F]FLT target-to-background ratios. Therefore, when the target tumor is present in these organs, [^18^F]FLT PET should be employed with caution [112].

Furthermore, it was shown that in patients with metastatic breast cancer who carry the BRCA mutation, [^18^F]FLT uptake correlates with their response to treatment with an oral poly-ADP-ribose-polymerase (PARP) inhibitor (veliparib) in conjunction with carboplatin and paclitaxel [113].

#### 3.9.3. HER2-Targeted Agents

The multicenter ZEPHIR study proposed the use of [^89^Zr]trastuzumab (HER2-PET/CT) to detect lesions in patients with HER2-positive metastatic BC and showed that pretreatment imaging using HER2-PET/CT is a viable method for examining interlesion heterogeneity in advanced disease stages. Following a cycle of trastuzumab emtansine and with the combined use of early [^18^F]FDG PET/CT, a group with a better outcome (median TTF of 15 months, with a positive HER2-PET/CT associated with a response on early [18F]FDG PET/CT) was distinguished from a group with a worse outcome (median TTF of 2.8 months when a negative HER2-PET/CT preceded a non-response on early [^18^F]FDG PET) [114,115].

Inhibiting receptor dimerization and subsequent signaling pathways, pertuzumab is a single-epitope monoclonal antibody that binds to HER2 at a single extracellular domain [116]. Zirconium 89-pertuzumab PET/CT, which targets HER2, may identify candidates for HER2-targeted therapy who would not otherwise be considered [117].

#### 3.9.4. FAPI

A fibroblast activation protein (FAP) inhibitor labeled with gallium 68 (^68^Ga) (FAPI) PET is based on FAP’s molecular targeting, which is known to be highly expressed in cancer-associated fibroblasts, the main cell type in the tumor stroma. This radiotracer has been tested in six ILC patients at baseline and after treatment (2–4 follow-up scans), revealing a strong correlation between FAPI tumor volume and blood biomarkers (r = 0.7, *p* < 0.01) [118]. PET/CT has also proven to be useful in monitoring the effectiveness of [(^177^)Lu]Lu-DOTAGA.FAPi dimer radionuclide therapy [119].

#### 3.9.5. Immunotherapy

Using the SP142 immunohistochemical assay as a gold standard, a first-inhuman study with 22 patients (including 4 patients with TNBC) demonstrated a high correlation between [^89^Zr]atezolizumab uptake and treatment response, PFS, and OS [120]. In a study assessing TNBC patients receiving neoadjuvant chemotherapy (NAC) or chemoimmunotherapy (NACI with pembrolizumab), researchers used [^18^F]FDG PET/CT scans before treatment to predict pathological complete response (pCR). They analyzed data from 191 patients and found that high tumor metabolism (measured by SUVmax) and low tumor burden (measured by TMTV) were predictive factors for achieving pCR after treatment, regardless of whether pembrolizumab was included [121].

#### 3.9.6. PET/MRI

Backhaus et al. examined the effectiveness of [^68^Ga]FAPI PET/MRI in determining the response of local BC and lymph node metastases after chemotherapy. Thirteen women who finished chemotherapy for invasive BC underwent [^68^Ga]FAPI PET/MRI and fibroblast activation protein (FAP) immunostainings. The authors compared FAPI uptake and FAP immunoreactivity between those who achieved a complete response (pCR) and those who did not. The results showed a significant correlation between FAP immunoreactivity and response status. [^68^Ga]FAPI PET/MRI successfully differentiated response statuses in BC, whereas MRI alone had some false-positive outcomes. For lymph nodes, PET/MRI had two false-negative readings; MRI alone had two false-negative readings and one false-positive result. The study suggests that [^68^Ga]FAPI PET/MRI might offer improved diagnostic capabilities compared to MRI alone, although larger studies are needed for confirmation [122]. Another study aimed to evaluate the effectiveness of [^18^F]Fluoroethylcholine ([^18^F]FEC) in PET/MRI imaging for assessing breast lesions and cancer aggressiveness and predicting lymph node status. The prospective single-center study enrolled women with suspicious breast lesions, employing histopathology as a reference. Radiologists and nuclear medicine physicians jointly analyzed MRI-detected lesions, measuring [^18^F]FEC uptake in the breast lesions (SUV(maxT)) and the axillary lymph nodes (SUV(maxLN)). The authors found an ROC value of 0.846 for distinguishing benign from malignant breast lesions. Higher SUV(maxT) values correlated with malignancy, increased proliferation rates, and HER2-positive lesions. Elevated SUV(maxLN) values were associated with metastatic lymph nodes, showing ROC values of 0.761 and 0.793 for SUV(maxT) and SUV(maxLN), respectively. The study concluded that simultaneous [^18^F]FEC PET/MRI is safe and holds promise for assessing breast cancer aggressiveness and predicting lymph node status [123].

#### 3.9.7. Radiomics

Radiomics explores intricate attributes within medical images to gain deeper insights into tumors. Meanwhile, machine learning involves instructing computers to comprehend these data, enabling them to detect patterns and forecast outcomes in fields like disease diagnosis and treatment planning by leveraging these image characteristics. Their combined use presents an avenue to extract valuable insights from images, enhancing medical decision-making processes. Some authors aimed to explore the effectiveness of using radiomics and machine learning with PET/CT images to predict HER2 status in BC. They studied 217 patients, splitting them into training and testing groups. Four machine learning models were tested, with the XGBoost model showing the best performance in predicting HER2 status. Particularly, combining PET/CT radiomic features significantly enhanced the accuracy of HER2 status prediction compared to using PET or CT alone. The findings suggest that a machine learning classifier utilizing PET/CT radiomic features has potential for predicting HER2 status in BC [124]. Similarly, in 2023, another study aimed to assess the effectiveness of radiomic signatures derived from [^18^F]FDG PET/CT images in determining HER2 status in primary BC cases where immunohistochemistry (IHC) results for HER2 were inconclusive. They analyzed 154 cases and compared conventional PET parameters between an HER2-positive and an HER2-negative group, finding them ineffective in distinguishing between the two. However, the radiomic signature model they developed showed promise, achieving an AUC of 0.887 in the training cohort and 0.766 in the validation cohort. External validation yielded an AUC of 0.788, suggesting the radiomic signature’s potential for predicting HER2 status non-invasively, particularly in cases with uncertain IHC results [125]. Other researchers sought to explore the potential of predicting PFS in BC patients using a combination of pretreatment [^18^F]FDG radiomic signatures and clinical parameters. The study included 87 patients, randomly divided into training, internal test, and external validation sets. Clinical data, including age, tumor size, molecular subtype, clinical TNM stage, and laboratory findings, were collected alongside radiomic features extracted from preoperative PET/CT images. The predictive radiomic signature, clinical model, and their integrated form (ICR model) were assessed for their associations with PFS using statistical models and nomograms. The ICR model, combining clinical parameters and PET/CT imaging features, demonstrated significant predictive capabilities for PFS in both the training and test sets, outperforming the clinical model and radiomic signature alone. External validation supported the robustness of the ICR model, indicating its potential to independently predict PFS in BC patients, surpassing the predictive abilities of the clinical model and radiomic signature in isolation [126].

## 4. Conclusions

In recent years, there has been a proliferation of studies on the use of PET in the assessment of breast cancer treatment. Thus, the utility of [^18^F]FDG PET/CT in the initial staging, restaging, evaluation of treatment response, and prediction of the prognosis of BC is now confirmed by a substantial amount of data [29,127].

The presented studies show the main evolutionary lines of PET: technological innovations (PET/MRI and PET scanners dedicated to the breast) and the development of new radiopharmaceuticals increasingly linked to the biological characterization of the various subtypes of BC. Promising fields of use of PET imaging include the assessment of responses to immunotherapy, PET/MRI, radiomics, and machine learning.

The large number of studies produced in recent years on the evaluation of responses to therapy in BC would justify, in our opinion, a greater consideration of PET/CT in international guidelines.

## Figures and Tables

**Table 1:** Distribution of selected studies based on the year of publication.

Year	Up to 2017	2018	2019	2020	2021	2022	2023
Studies	30	25	16	24	24	1	7

**Table 2:** EORTC and PERCIST criteria.

Response Criteria	EORTC	PERCIST
Imaging Modality	CT (Computed Tomography), MRI (Magnetic Resonance Imaging), bone scan	PET (Positron Emission Tomography)
Lesion Assessment	Lesion sizes (uni-/bi-dimensional)	FDG (Fluorodeoxyglucose) uptake
Response Classification	Complete Response (CR), Partial Response (PR), Stable Disease (SD), Progression (PD)	Complete Metabolic Response (CMR), Partial (PMR), Stable (SMR), Progression (PM)
Parameters	Measurement of lesion sizes in mm	FDG uptake and distribution in active lesions normalized to healthy tissues
Progression Evaluation	Increase in lesion diameter, new lesions	Increase in metabolic activity of lesions; new metabolically active lesions
Utility	Mainly focuses on evaluating tumor lesion sizes	Focuses on changes in metabolism in tumor lesions

**Table 3:** List of some of the main PET radiotracers used to image breast cancer.

Radiotracer	Radioisotope	Target	Characteristics
^18^F-FDG	F-18	Glucose uptake	Increased uptake in highly metabolic tissues
^18^F-FES	F-18	Estrogen receptor	Binds to estrogen receptors in cancer cells
^18^F-FLT	F-18	Cell proliferation	Marks proliferating cells
^18^F-NaF	F-18	Bone metastases	Used for detecting bone metastases
^89^Zr-Trastuzumab	Zr-89	HER2 receptor	Targets HER2-positive breast cancer
^18^F-Fluciclovine	F-18	Amino acid transport	Used for imaging amino acid transport in tumors
^68^Ga-FAPI	Ga-68	Fibroblast Activation Protein (FAP)	Targets FAP overexpressed in cancer-associated fibroblasts
^18^F-Fluoroethylcholine	F-18	Cellular membrane integrity	Used for imaging cellular membrane turnover
